# Long-lasting effects of incentives and social preference: A public goods experiment

**DOI:** 10.1371/journal.pone.0273014

**Published:** 2022-08-25

**Authors:** Maho Nakagawa, Mathieu Lefebvre, Anne Stenger

**Affiliations:** 1 BETA - University of Strasbourg, Strasbourg, France; 2 Chair of Environmental Economics, Brandenburg Technical University Cottbus-Senftenberg, Cottbus, Germany; 3 Aix Marseille Univ, CNRS, AMSE, Marseille, France; 4 BETA, INRAE, University of Strasbourg, Strasbourg, France; Teesside University, UNITED KINGDOM

## Abstract

This paper addresses the question of the effectiveness and permanence of temporary incentives to contribute to a public good. Using a common experimental framework, we investigate the effects of a recommendation that takes the form of an exhortative message to contribute, a monetary punishment and a non-monetary reward to sustain high levels of contributions. In particular, we shed light on the differential impact these mechanisms have on heterogeneous types of agents. The results show that all three incentives increase contributions compared to a pre-phase where there is no incentive. Monetary sanctions lead to the highest contributions, but a sudden drop in contributions is observed once the incentive to punish is removed. On the contrary, Recommendation leads to the lowest contributions but maintains a long-lasting impact in the *Post-policy* phase. In particular, it makes free-riders increase their contribution over time in the post-incentive phase. Finally, non-monetary reward backfires against those who are weakly conditional cooperators. Our findings emphasize the importance of designing and maintaining incentives not only for free-riders, but for strong and weak conditional cooperators as well, depending on characteristics of the incentives.

## Introduction

An important issue for public policy in social dilemmas situations is how to design incentive instruments that are effective in changing individual behaviors. This explains why the use of behavioral interventions has become more and more popular. Although these interventions often take the form of financial incentives, their applications are still highly debated and, in the search for cost-effective policies, there is a growing interest in figuring out what are the best interventions. Indeed, [1, 2] have shown how important it is that incentives to cooperate are designed such as optimizing the overall cost of their implementation. In particular [2], provide a framework to determine optimal cost when monetary punishments are used. Given these cost-effectiveness considerations, non-monetary incentives are more and more discussed in the desired achievement of pro-environmental or pro-social behaviors [3]. They have been shown to increase voluntary contributions to public goods and induce pro-social behavior [e.g. 4–7]. In particular, there is evidence both in the lab and in the field, that priming and informational nudges perform well in inducing contributions to public goods [e.g. 8–11]. According to [12], “a nudge, […], is any aspect of the choice architecture that alters people’s behavior in a predictable way without forbidding any options or significantly changing their economic incentives” [12, p.6]. A nudge can then take various forms, and [13] provides examples such as default option, simplification, use of social norms, warnings and disclosure policies. In addition, there is an increasing interest in identifying the long-term effect of short-term public policies. [14] has pointed out that it is also important to go beyond the study of short-term effects in order to understand the effectiveness of incentives and, more globally, the implications for policy design. Whereas many incentive programs reveal short-term effects, evidence concerning long-term effects is often much more limited.

These questions are particularly important in environmental policy for which the definition of incentive contracts that promote cooperative behaviors has been challenged by the search for costless mechanisms. Moreover, since the environmental programs are often temporary for political and/or budgetary reasons, their effectiveness in the long run, i.e. after the programs have ended, is an issue. Well-known examples of this type of program are the agricultural environmental policies for biodiversity conservation, such as the Agri-Environment Schemes (AES) or the Payment for Ecosystem Services (PES). Most of the time, the protection of natural habitats requires a long-term commitment in a cooperative manner, but many payment schemes to compensate for pro-environmental practices are finite and individual contracts. As [15] point out, the research in behavioral economics lacks the specific testing of long-term effects of incentive programs, especially non monetary ones, in agri-environmental policy settings.

In this paper we report on a series of lab experiments designed to assess the effectiveness and the long-lasting effects of different temporary incentive schemes on contributions in a repeated public goods game. We use a linear voluntary contribution mechanism played by groups of four subjects in a fixed-partner and between-subject design to see how the introduction of different incentives affects contributions. In our experiment, subjects first play five periods of a standard public goods game. We then introduce an incentive to contribute for ten successive periods. Depending on treatment conditions, the incentive can be either a *Recommendation*, a *Non-monetary Reward* or a *Monetary Punishment*. Finally, incentives are removed and we then look at their long-lasting effect for a subsequent 15 periods.

Our experimental design aims at reflecting some sort of real-world setup wherein numerous incentive programs are only implemented for a given short amount of time. We aim at testing the relative efficiency of well-known programs in the field. The *Monetary Punishment* is similar to PES programs that have been recently implemented, i.e., the payment is reduced in accordance with the ratio of the total deforestation area [16]. The search for social approval or better social status through farm management practices has also been encouraged in AES [17, 18]. Our *Non-monetary Reward* attempts to mimic the effects of the quest for social approval that may stimulate the creation of a social norm. And *Recommendation* such as priming contribution through the use of an informational nudge has become more and more common in environmental and energy conservation programs [9, 14, 19].

There is an extensive experimental literature on voluntary contributions in social dilemma situations and how to increase them. See, in particular, the surveys by [20, 21] on the effect of various types of incentives to increase contributions to public goods. Although free-riding is a dominant strategy, a major result is that, in a finitely repeated public goods game, participants generally exhibit initial positive contributions that gradually decrease over rounds. The interaction between conditional cooperators and free-riders has been pointed out as an explanation for this result. Conditional cooperators are willing to contribute only if the other group members also contribute, and almost always form a majority of the population. The presence of free-riders that do not contribute make conditional cooperators reduce their contributions once they interact in the same group and, thus, reduce the total contributions of the group over rounds [22].

Previous experiments have shown that introducing monetary incentives such as formal sanctioning increases contributions and slows down the decay observed with repetitions [see, e.g., 23, 24]. However recent works by [25, 26] have shown that the effect of punishments on cooperation can be widely affected by corruption. As [26] point out, it cannot be ignored that punishment is vulnerable to corruption. When there is a possibility to bribe, it undermines the effectiveness of punishment on promoting cooperation. Non monetary incentives have also been shown to be effective to sustain cooperation. In particular, [4, 6] point out that the possibility of subjects’ approval and disapproval ratings in a linear public goods game significantly increases the contributions. [5, 27] compare the effectiveness of non monetary sanctions and rewards and find that both increase the individual contributions, whereas sanctioning appears to be a more effective mechanism for sustaining a high level of cooperation. [28] perform a meta analysis on rewards and punishments and conclude that they are both effective in promoting cooperation in social dilemmas. One channel through which non monetary rewards and punishments are effective is reputation [29]. There is also an increasingly abundant literature that has shown the effectiveness of persuasive messages, priming and moral nudges on voluntary contributions. Since individuals’ attitudes toward contributions are affected by social interaction through subjective norms, behaviors may be modified by introducing normative statements that act on intentions [30]. Lab experiments have demonstrated that priming can be effective in increasing contributions to a public good [31]. [32–34] show that introducing moral messages could have a positive and significant effect on contributions. By priming people about what behavior is expected and appreciated, one introduces a kind of social pressure. People then incur a psychological cost on their social image if they deviate from the informational message [11, 35]. See also [36] for a survey of theoretical and experimental evidence on general moral preferences, According to the so-called *moral preference hypothesis*, people have preferences for following their personal norms, beyond the economic consequences that these actions bring about. As they pointed out, when people care about doing the right thing, then just reminding them of the rightness of an action can work in promoting desirable behaviour.

Although understanding the extent to which those policies have persistent effects is important, there is little literature on this question. Identifying which channel is the driver of change, if any, is a difficult task, and there are several reasons why an incentive program could have long-lasting effects. They can be due to, i.e., an information update [37], the adoption of new habits [27] or new social norms [9]. Recent empirical papers have focused on the persistence of the impact of an incentive programs once it is suspended, and the results are mixed. [33] show in an experimental design that an exhortative message appealing to participants’ goodwill can increase cooperation in social dilemmas played over many rounds. Comparing different social dilemmas situations, [38] demonstrate that policy interventions such as push measures (rebates and minimum donation rules) are more effective than nudges, and that their effects are persistent over time, but only when the context remains the same over time. In a minimum effort game, [39] explore the effect of introducing incentives for effort and cooperation, and the effect of a subsequent removal of these incentives. They find that reductions in the incentives have little effect on subsequent behavior. On the contrary, in a similar design, [40] find few persistent long-term effects, with the effort reverting back to its pre-incentive level. In a repeated public goods game, [41] investigate whether temporary monetary incentives to contribute have an impact on later periods. Their results show no persistent effects, and furthermore, cooperation rapidly deteriorates to levels lower than those of the control groups.

On the grounds of this evidence, the originality of our paper is threefold. First, in a repeated public goods game in a fixed partner design, we compare the effectiveness of three different incentives that are increasingly discussed in environmental policy: a *Monetary Punishment*, a *Non-monetary Reward* and a *Recommendation*. While the effect of similar mechanisms has been documented, no study has yet to compare their relative effectiveness in a common framework. We distinguish *Recommendation* and *Non-monetary Reward* even though both can be placed in the same category of “non-monetary incentive mechanisms”. This is because the participants only passively receive a message in *Recommendation*, whereas *Non-monetary Reward* requires an active effort on the part of the participants. Second, we look at the long-lasting effects of those incentives when they are removed after a fixed number of periods. This is done in a within and a between design at the same time. Finally, a third originality of our paper is that we elicit the subjects’ social preferences, using the so-called Strategy Method [22]. This allows us to classify subjects by preference types as free-riders, strong or weak conditional cooperators, and to compare their behavior against the incentive. Moreover, we elicit subjects’ preferences before and after the repeated game to identify possible inconsistencies between individuals’ actual contributions and their preferences. This is important to understand how types of contributors are differently affected by the incentives. In the field, [19] reveal that farmers participating in the French AES scheme are generally conditional cooperators. Moreover, [42] suggest that the share of conditional cooperators in the group is a criterion of success in forest commons management. Policymakers need to determine which incentives will be effective for people with certain preferences in order to select policies that work well for the target population.

Our results show that all incentive mechanisms significantly increase contributions in the short run. The highest average contribution is observed in *Monetary Punishment* during the treatment phase, followed by *Non-monetary Reward* and *Recommendation*. Notably, *Monetary Punishments* significantly increase the contributions, regardless of the types of subject, but this increase is followed by a sudden drop once the incentives are suspended. Although we generally observe similar long-lasting effectiveness in the post-intervention periods, the treatment conditions have diverging effects depending on the preference types of subjects. The *Recommendation* treatment has a long-lasting impact on those who strongly and conditionally cooperate with others. It also makes free-riders increase their contribution but ends up with the lowest average contribution on average across treatments. The *Non-monetary Reward* condition backfires on those who are weakly conditional cooperators. On the contrary, the types of subjects seem to matter less in *Monetary Punishment*.

The paper is organized as follows. Section describes the experimental design and the procedures. Our predictions are shown in Section. Section presents the results, and Section includes a discussion and a conclusion.

## Materials and methods

### Experimental design

The experiment is broken down into three successive parts. First, we elicit the preference for cooperation through a public goods game played in Strategy Method mode. Then, the subjects play the main task, which consists of a repeated public goods game (PGG). Finally, they play the public goods game once again in Strategy Method mode to check the consistency of preferences.

### Main task

The main task consists of a finitely-repeated public good game played for 30 periods by fixed groups of four subjects. At the start of each period, each subject receives an endowment of 20 tokens and has to simultaneously decide how many tokens to invest into a public account. Each token invested in the public account yields 0.4 tokens for each member of the group. Subjects keep the tokens they do not invest for themselves. Therefore, in each period, the earnings of individual *i* who contributes *c*_*i*_ to the project are given by:

$$\begin{array}{c} \pi_{i}=20-c_{i}+0.4\sum_{k=1}^{4}c_{k} \end{array}$$ (1)

Subjects are informed of the contribution levels of other members after each period. However, individual decisions are not linked to subject identifiers and contributions are presented in ascending order in each period so that subject-specific reputations cannot develop across periods.

We consider three different conditions in which we vary the incentive schemes to contribute: either a *Non-monetary Reward*, a *Monetary Punishment* or a *Recommendation*. The 30 periods of the repeated game are broken down into three successive phases. In all three conditions, subjects start by playing five periods of the standard PGG with no incentives to contribute. This is the *Pre-policy* phase. During this phase, they do not know that in subsequent periods, they will face incentives. Thus this *Pre-policy* phase is identical in all three treatment conditions. Then, depending on the treatment condition, they face an incentive mechanism for 10 periods. This is the *Policy* phase. Finally, a *Post-policy* phase ends the game with 15 periods of the standard PGG again with no incentives. Table 1 provides the basic design information, and instructions for *Monetary Punishment* are presented in S1 Appendix.

**Table 1:** Treatment conditions.

	Subjects	*Pre-policy* (Periods 1–5)	*Policy* (Periods 6–15)	*Post-policy* (Periods 16–30)
Monetary Punishment	40	PGG	PGG + Punishment	PGG
Non-monetary Reward	40	PGG	PGG + Reward	PGG
Recommendation	40	PGG	PGG + Recommendation	PGG

In the *Policy* phase (Periods 6–15), the subjects face different incentives to contribute according to the treatment condition:

(i) In the *Monetary Punishment* condition, subjects go through two stages. First, they decide on their contribution. Second, after being informed of the contribution levels of each of the other members of their group, subjects can assign zero to 10 punishment points to each of the three other group members. Each point, *p*_*ij*_ assigned by subject *i* to subject *j*, lowers subject *j*’s income by one token. There is also a cost of 0.25 tokens for subject *i* associated with each point allocated. The effectiveness of the punishment mechanism has been shown to be related to the mix of cost-impact of the punishment. [43] show that a low cost-high impact punishment is the most effective mechanism. We opted for a 1 to 4 ratio. We also present each member’s individual income instead of their contributions. However, [44] has shown that giving the individual income instead of the individual contributions reduces the effectiveness of the punishment mechanism. The choice of punishment points is restricted to the actual earnings from the first stage, but the earnings at the end of a period can be negative depending on the number of punishment points distributed and received. This implies that payoffs at the end of second stage, and therefore, for the given period are given by:
  $$\begin{array}{c} \Pi_{i}=\pi_{i}-\sum_{j\neq i}p_{ij}-0.25\sum_{j\neq i}p_{ji} \end{array}$$ (2)
(ii) In the *Non-monetary Reward* condition, subjects can also assign zero to 10 points to each of the other group members. However, these points have no effect on the the subjects’ final earnings and are assigned at no cost. They are meant to express the subject’s approval of the group’s members’ contributions. Zero points correspond to the lowest level of approval and 10 points to the highest level of approval.
(iii) Finally, in the *Recommendation* condition, in each of the Periods 6–15, before deciding to contribute or not to the public good, a message informing subjects about socially-optimal contributions is displayed. The message is enclosed in a blinking dot frame and shown in red. In addition, the subjects cannot proceed to the next step unless they click on an acknowledgement button:
*The best contribution for you and your group is that*
  *each one contributes 20 tokens to the public account*.
  Subjects are thus reminded of the best outcome when all four members contribute. We cannot reject the possibility here of a demand effect [45], but none of the experimenters involved in any of the sessions is a professor at the university.

In the *Non-monetary Reward* and *Monetary Punishment* treatments, subjects are informed of their earnings at the end of each period as a result of assigning or receiving points. Information about the total number of points is also provided, but the subject cannot identify which member of the group assigns her points. Moreover, subjects are not informed of the points received by other group members.

In Periods 16–30 (the *Post-policy* phase), there is no more incentive intervention and the task is identical to the one in Periods 1–5. The end of the incentives is clearly stated in the instructions provided at the beginning of Period 6 in all treatment conditions. In addition, subjects know that the experiment consists of a finitely repeated game with a final 30th period.

In each of the 30 periods of the experiment, we elicit beliefs about the other group members’ average contribution. We follow the example of [46] and ask subjects to enter their contribution decisions as well as their estimation on how much they think the other three group members will contribute on average to the public good. A strong positive or negative correlation between belief and contribution implies that beliefs are a key to explaining the different effects of incentives. For the sake of eliciting accurate beliefs and avoiding the possibility that subjects project their own contribution onto their beliefs about others’ behavior, we incentivize the task through a nonlinear scheme, as in [46]. The instructions are presented in Task 2 in S1 Appendix.

### Preference elicitation

As explained above, we elicit preferences for contributions before and after the main task by using a one-shot public goods game played in the Strategy Method [22]. The instructions for the Strategy Method are presented in Task 1 in S1 Appendix. This is done to elicit subjects’ preferences and to check for consistency throughout the experiment. The task is in all respects similar to the one described in the main task for the repeated PGG, except that it is clearly a one-shot game. Subjects are asked to make two decisions. First, they choose a contribution level in a one-shot public goods game: the ‘unconditional contribution’. Then, they are asked to fill in a contribution table that contains the 21 possible average group contributions from zero to 20. They have to select a contribution level for every possible average contribution of the other group members. In each group, earnings are computed using the unconditional contributions of three randomly-selected subjects and the contribution table of the fourth one. The composition of the group is randomly drawn each time and different than in the main task.

Like in [22, 47, 48], we use the results from the first Strategy Method (referred to as 1st SM hereafter) to classify subjects into preference types. We define four types: free-rider, strong conditional cooperator, weak conditional cooperator, and “other” type:

(i) Free-rider: If the subject’s average contribution for the 21 entries is below 10% of the endowment.
(ii) Strong conditional cooperator (Strong CC): If the subject raises her own contribution when the others’ average contribution is increasing with the correlation between her own contributions and the others’ average, which is above 70%. In addition, her total contributions for the 21 entries is within ±30% of the contribution of a perfect conditional cooperator.
(iii) Weak conditional cooperator (Weak CC): If the subject raises her own contribution when the others’ average contribution is increasing but the link is less strong. In particular, a subject is categorized as a weak conditional cooperator if Spearman’s rank correlation between her own contributions and the others’ average is positive and significant at the 1%-level.

In the event that a subject does not match any of the above-mentioned type classifications, we categorize the subject as “other” type. Our classification follows more or less [48]. We introduce a new “weak conditional cooperator” status that allows us to differentiate by degree of cooperation in the results. As pointed by [48], while the chosen thresholds may appear arbitrary, they allow to identify clearly distinct types. See also [49]’s meta-analysis of preference types in public good games who adopt a similar procedure.

### Procedures

In total, 120 subjects participated in six sessions (two sessions per treatment) at the Laboratory of Experimental Economics of Strasbourg (LEES) in November 2017. All participants were recruited from a list of experimental subjects maintained at the LEES, using the ORSEE software [50]. Subjects had an average age of 21 years (standard deviation = 3.31), and 53% of the subjects were female. They were from different fields, but 32% were studying economics or business management.

The experiment was computerized using EconPlay. EconPlay was designed by Kene Boun-My (www.econplay.fr). Upon arrival, each subject was randomly assigned to a computer. The instructions were read aloud by an experimenter, and a comprehension questionnaire was administered to check that the rules were well understood before starting the experiment. If a subject had two or more false answers, an experimenter came to explain and answer questions. The subject could not continue to Task 1 before solving the questionnaire. All questions were answered in private. After the three tasks were completed, the screen displayed the total cumulative gain for the experiment and the subject answered a post-experiment questionnaire. Then, at the end of the session, subjects were paid their earnings. The conversion rate was 50 tokens to 1 euro for PGG and 10 tokens to 1 euro for the Strategy Method. The average earning was 20.81 euros (standard deviation = 2.21). Sessions lasted between 60 to 90 minutes.

All participants had previously given formal written consent to be part of the experiment. Anonymity was guaranteed and thus the analysis was done on anonymized data. No Ethic Committee at the University of Strasbourg had to approve the experiment.

## Predictions

Assuming that subjects care only about monetary payoffs, are fully rational and that it is common knowledge, they should not contribute in the *Pre-policy* phase since free-riding is a dominant strategy. In contrast, the social optimum is achieved when all of the subjects in a group choose to fully cooperate by contributing all of their endowment to the group account. We know from the extensive literature on public goods games that we can expect positive contributions in the absence of incentives followed by a continuous decay due to the presence of conditional cooperators [21].

This unstable cooperation has been shown to be rectified by the introduction of *Monetary Punishment* [4, 5, 23]. Subjects are willing to engage in the punishment of free-riders, and the possibility of being sanctioned should lead to higher contributions. Previous studies have also shown that *Non-monetary Rewards* lead to higher and more stable contributions [6, 51, 52]. Although a meta-analysis of [28] reveals that the relative effectiveness of punishments and rewards in a repeated game does not statistically differ in the impact on cooperation, the closest experimental design to ours shows that a *Monetary Punishment* outperforms a *Non-monetary Reward* reward [27]. Although monetary incentives are also well known to crowd out individuals’ motivation to contribute voluntarily (see [53–55]), monetary punishments have been shown to be effective in public good games.

There is also evidence to suggest that persuasive messages are successful in enhancing cooperation. A message exhorting contribution may affect participants’ preferences by raising the level of contributions they deem to be appropriate and thus raising the utility weight on meeting that level [32]. The message may also change participants’ expectations about others by raising optimism about how their fellow subjects might contribute [22, 33, 56]. However, this effect has been shown to be dependent on how participants value the public good and on the specific content of the message [57, 58]. [59] point out that the impact of information relies on the disposition of people to be involved into a long-run perspective or on the framing of the situation. Moreover, [33] demonstrate that an exhortative message appealing to participants’ goodwill to contribute to the public good leads to higher contributions than a punishment treatment, particularly when group composition is fixed over time.

**Prediction 1**. *All three treatment conditions should lead to higher contributions in the* *Policy phase than in the Pre-policy phase but we can expect that a) contributions are higher in Recommendation than in Monetary Punishment and b) contributions are higher in Monetary Punishment than in Non-monetary Rewards*.

Each incentive appears to have its own channel in exerting the effect, that is, their effectiveness relies on the disposition of people. *Monetary Punishments* are effective because, in encouraging free-riders to contribute, they consequently push the conditional cooperators to maintain their level of contributions. In particular, strong CCs may react positively to this higher level of contributions in the group. This reduces the overall decay of contribution. The effect of *Non-monetary Rewards* may also depend on agent’s social preference. In the context of voluntary conservation programs, [18] find that non-monetary incentives, taking the form of a facilitation service with social reward, affects mainly pro-environmental farmers. The expected positive average effect of *Non-monetary Rewards* would thus be the result of higher contributions by conditional cooperators, who are in our case, strong CCs. Finally, *Recommendation* may also be more effective for those who actually are open to receive this reminder. In a contextualized experiment (an environmental public goods game) in which they also elicit subjects’ environmental sensitivity, [35] show that an informational nudge may result in a lower contribution during the intervention by crowding out free-rider’s motivation to undertake an action. Relying on individuals’ environmental sensitivity, they show that suggesting to an agent to act in a given direction (such as investing in the environmental account in their experiment), when this agent has little interest in the concerned outcome, would reinforce the lack of concern of this agent in the outcome.

**Prediction 2**. *Looking at individual social preferences: a) Monetary Punishment should lead to higher contributions by both free-riders and conditional cooperators. b) Non-monetary Rewards and Recommendation should only affect cooperators*.

Whether these three incentive mechanisms have long-lasting effects once they are removed in the *Post-policy* phase is not clear-cut. The impact will depend on the participants’ intrinsic nature [60] and how it changes subjects’ behavior by affecting their subjective norms. Especially, [41] show that even after the removal of incentives, predictions about contribution levels depend on the individual preferences. If we assume that the incentives primarily influence contributing behavior, contribution should go down to the baseline. On the contrary, if we assume that the incentives improve coordination and perhaps even create trust and self-image that should influence later interactions, we should observe a high level of contributions in the Post-treatment phase [39]. Image concern has been shown to be a reason to maintain high average contributions even when strong material incentives have been removed [61].

The incentives can have different long-lasting effects. A number of studies suggest that the contribution levels decrease to a level below the baseline in *Monetary Punishment* due to the crowding-out of intrinsic motivation [41, 53, 55, 62, 63]. *Monetary Punishment*, because of its versatility in that it can have positive effects on both free-riders and strong CCs during implementation, may be more likely to backfire on both types later. [6, 64] provide the evidence that *Non-monetary Reward* is not sustainable and has only a short lived effect. It is conceivable that *Non-monetary Reward* could also backfire due to the frustration that efforts to reward others do not pay off, but to the best of our knowledge, we have not found find any literature that showed this type of results under the experimental design similar to ours. Greater persistent effects with *Recommendation* can be expected if the recommended message changes individuals’ attitude toward some situation. Once this attitude is changed, it may be expected to last for a while [9, 65]. This does not mean that there will be no adverse effect through the disutility of those who has a low valuation of public goods [35], but free-riders are generally not the majority.

**Prediction 3**. *Although all three treatment conditions should reduce the decay of contributions in the Policy phase, they should have diverging long-lasting effects in the Post-policy phase: a) Greater persistent effects are expected from Recommendation than the other two treatments, and b) conditional cooperators (weak or strong) should maintain contributions much longer than other types of subjects*.

## Results

Our results are presented as follows. First, we present the contributions in the main task and show the effects of our three incentive mechanisms on their levels. We then explore the heterogeneity in subjects’ cooperation preferences and whether or not it explains their contribution patterns.

### Group contributions

On average, subjects contribute a total of 40% of their endowment. If we separately look at each treatment condition, the highest level of contribution is achieved by *Monetary Punishment* with 48% of the endowment, while we observe 32% and 39% in *Recommendation* and *Non-monetary Reward*, respectively. Those numbers need to be disaggregated at the phase level to understand the different effects of incentives. Table 2 presents the average contributions by group with the standard deviation in each treatment and between phases.

**Table 2:** Average contribution.

	Pre-policy (Periods 1–5)			Policy (Periods 6–15)			Post-policy (Periods 16–30)		
	Mean	Std.Dev.	Obs	Mean	Std.Dev.	Obs	Mean	Std.Dev.	Obs
*Group average*									
Recommendation	19.86	10.11	50	35.07	22.22	100	20.47	20.48	150
Non-monetary Reward	21.52	17.10	50	43.06	22.55	100	27.15	25.07	150
Monetary Punishment	21.48	14.75	50	59.02	23.35	100	30.53	32.47	150

Unit(token)

**Fig 1:**
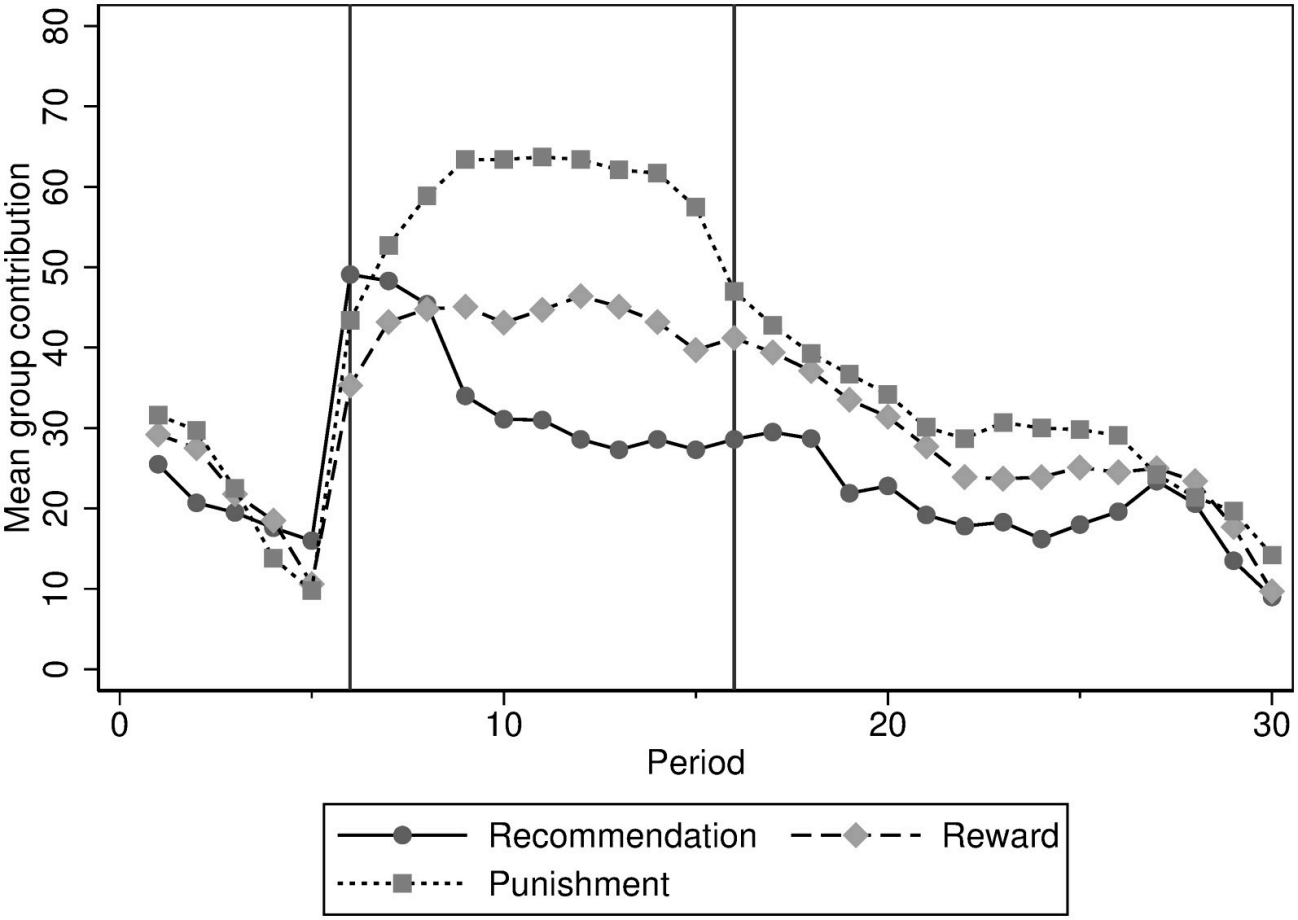
Average group contribution.

According to a Kruskal-Wallis test that takes group averages as the unit of observation, the average contributions during the *Pre-policy* phase do not differ between the three treatment conditions (*χ*^2^(2, 150)=0.06 *p*=0.97). Our tests, with 10 independent observations in each treatment, were at risk of being underpowered if the effect size is not large. Indeed, a post-hoc power analysis using G*Power [66] is conducted and we find out that our tests fall short of power relative to an acceptable level of 0.8. However, we also conduct regression analyses in the next subsection with panel data to deal with our limitation. The similar pattern of contribution during the *Pre-policy* phase can be seen in Fig 1, which illustrates the average group contributions for each period in the three treatments. The two vertical lines at Period 6 and Period 16 define the division among the three phases. The absence of difference during the *Pre-policy* phase confirms that there is generally no significant difference between the three samples of subjects.

In the first period, the average contribution is a little lower (36% of the endowment on average) than the one generally observed in other studies in which contributions usually range from 40 to 60% of the endowment [see 20, 21]. Although the contributions are initially low in the *Pre-policy* phase, they are similar to previous findings in short repeated public good games. [67] found an initial contribution of 35% of the endowment in a 5-period game. Other studies also introduced short-term no-incentive phases at the beginning of a repeated game and found an average contribution of about 40% of the endowment in the initial period [24, 43, 68, 69]. The rate of decay in the *Pre-policy* phase is immediate and large so that the average contribution ends up with only 15% of the endowment at Period 5. This may be due to the fact that participants know that this particular task lasts for only five periods. We cannot exclude the possibility of an end-of-the game effect that is likely to be stronger in this phase compared to other phases given the lower number of periods.

We then look at the effect of incentives introduced during the *Policy* phase. A Friedman test that takes group averages as the unit of observation displays significant differences between the three phases in each treatment condition (*Recommendation*: *Q*(2)=59.69 *p*<0.01, *Reward*: *Q*(2)=80.66 *p*<0.01, *Punishment*: *Q*(2)=104.40 *p*<0.01). This is confirmed by pairwise comparisons using Dunn’s post-hoc tests. For pairwise comparisons, we use Dunn’s multiple non-parametric pairwise tests [70, 71] instead of the Wilcoxon-Mann-Whitney test because it is relatively flexible and has a greater power, although the results are similar [72]. We apply Dunn’s test after Friedman (for dependent/within-subjects design) as well as the Kruskal-Wallis test (for repeated measures/between-subjects design). Tests show that there is a significant difference between the contributions in the *Pre-policy* and *Policy* phases in each of the three treatments (*z*-test statistics of tests of *Pre-policy* vs. *Policy* phases with Bonferroni adjusted *p*-values for multiple comparisons. *Recommendation*: *z*=-7.10 *p*<0.01, *Reward*: *z*=-9.73 *p*<0.01, *Punishment*: *z*=-12.6 *p*<0.01). This confirms Prediction 1: all treatment conditions increase contributions. However, we see in Table 2 and Fig 1, the three incentives do not have the same impact on contributions.

During the *Policy* phase, the highest level of contributions is achieved by *Monetary Punishment* followed by *Non-monetary Reward* and *Recommendation*. A Kruskal-Wallis test that takes group averages as the unit of observation rejects the null hypothesis of equality (*χ*^2^(2, 300)=51.66 *p*<0.01). Pairwise comparisons of treatments contributions using Dunn’s post-hoc tests show significant differences between every treatments (z -test statistics with Bonferroni adjusted p-values for multiple comparisons. *Recommendation* vs. *Reward*: *z*=-4.36 *p*<0.01, *Recommendation* vs. *Punishment*: *z*=-14.1 *p*<0.01, *Reward* vs. *Punishment*: *z*=-9.70 *p*<0.01). These results confirm the effectiveness of the three incentive mechanisms and confirm Prediction 1b since *Monetary Punishment* displays higher contributions than *Non-monetary Reward*. They contradict Prediction 1a since *Monetary Punishment* has higher contributions than *Recommendation*.

In the *Post-policy* phase, regardless of the treatment condition, the incentives to contribute are removed. As shown in Table 2, in each treatment, we generally observe a drop in contributions. A Dunn’s post-hoc test with Bonferroni adjusted p-values for multiple comparisons reveals that the difference in contributions between the *Policy* and *Post-policy* phases is significant in each treatment (*Recommendation*: *z*=10.97 *p*<0.01, *Reward*: *z*=10.49 *p*<0.01, *Punishment*: *z*=14.85 *p*<0.01). Interestingly, a comparison of contributions between the *Pre-policy* and *Post-policy* phases in each treatment only shows a marginal significant difference in the case of *Non-monetary Reward* (Dunn’s test z -test statistics with Bonferroni adjusted p-values for multiple comparisons. *Recommendation*: *z*=1.14 *p*=0.38, *Reward*: *z*=-2.02 *p*=0.06, *Punishment*: *z*=-1.64 *p*=0.15). This result suggests that all incentive mechanisms significantly increase contributions while they are in place, but that removing them could return contributions to the levels of *Pre-policy* on average, depending on the incentive characteristics. Moreover, during the *Post-policy* phase, the gap between treatments appears to narrow in Fig 1. A Kruskal-Wallis test confirms that there is no significant difference among treatments in the *Post-policy* phase at the 5% level (*χ*^2^(2, 450)=5.83 *p*=0.05). However, further investigation of individual contributions below will temper these results.

### Individual contributions

We now examine individual contributions in order to better explain the differences between treatments. In particular, we are interested in how the incentives reduce free-riding and increase full contributions.

**Fig 2:**
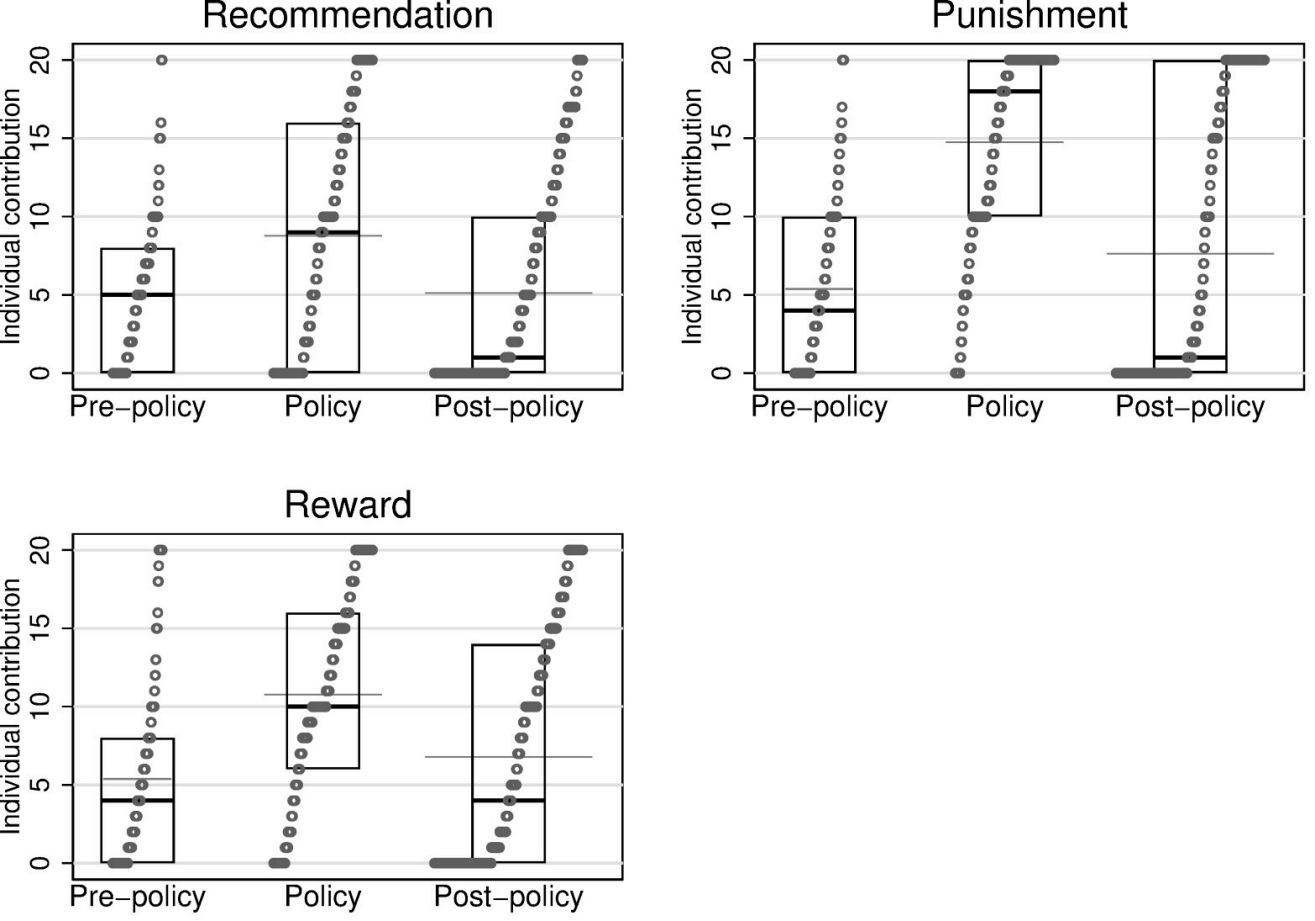
Quantile box plot for average individual contributions.

Fig 2 presents data plots for each phase in each treatment condition. In the figures, a box illustrates the median and the quantiles. The gray line represents the mean. Although the post-hoc tests on group averages suggest no evidence of a difference between the *Pre-policy* and *Post-policy* phases in each treatment, the variability of the data appears to be different. In particular, in *Monetary Punishment*, the contributions during the *Post-policy* phase are much more polarized, so that the box is longer than in the *Pre-policy* and *Policy* phases. This increase in the dispersion of observations is also true in *Non-monetary Reward*.

We investigate the differences in individual contributions across treatment conditions and policy phases by estimating Tobit and Probit regressions. First, we run Tobit estimations of the determinants of individual contributions in each treatment. The dependent variable is the individual contribution per period. Each specification includes control for age, gender and if the subject is studying economics. We introduce a dummy variable for time sequences, taking *Pre-policy* as the reference category. Since our design relies on a within-subject design for incentive effects, we do not have a specific baseline treatment that acts as a control group. Similar to our study, [27] present a between-subjects analysis of monetary and non-monetary incentives in a public goods game for 30 periods. In their baseline, they observe a significant and continuous decay of contributions. The tests of differences allow us to ensure that our *Policy* phases display significant differences with their baseline contributions (data are publicly available). We did not include a variable about subjects’ beliefs about others’ average contributions as an explanatory variable due to the strong correlation between belief and contribution. We run a Pearson’s correlation for each treatment. (*Recommendation*) r = 0.74 *p*<0.01; (*Reward*) r = 0.80 *p*<0.01; (Punishment) r = 0.90 *p*<0.01. This strong correlation can also be seen in Fig 6 in S3 Appendix. We also introduce a period variable and we control for the dependency of observations by clustering standard errors at the group level.

Results in Table 3 support our earlier statement that all incentives significantly increase contributions during the *Policy* phase compared to the *Pre-policy* phase. In addition, the highest contribution level is observed in *Monetary Punishment* followed by *Non-monetary Reward* and *Recommendation*. However, contrary to the results of non-parametric tests on group averages, contributions in *Post-policy* phases are significantly higher than in *Pre-policy* phases in all three treatment conditions once we control for covariates. Furthermore, in *Recommendation* and *Non-monetary Reward*, the incentives show persistent effects and the contributions are significantly higher in the *Post-policy* phase than in the *Policy* phase. The results suggest that the magnitude is higher in *Recommendation*. These results are driven by the effect of periods that take into account the diminishing trend of contributions, in particular in the Post-Policy phase. This suggests that for a given (common and hypothetical) period the contributions in the post-policy phase would be still high, all else being equal. According to the Wald tests shown in Table 3, the *Policy* and *Post-policy* phases are significantly different from each other in all treatment conditions. These results partially confirm our Prediction 3.

**Table 3:** Determinants of contribution.

Dependent variable:	Tobit estimation			Probit estimation					
	Individual contribution			Zero contribution			Full contribution		
	Reco	Reward	Punish	Reco	Reward	Punish	Reco	Reward	Punish
Policy (P6–15)	9.976 ***	12.268 ***	24.174 ***	-0.412 ***	-1.464 ***	-2.077 ***	1.705 ***	0.492 ***	1.773 ***
	(1.475)	(2.595)	(4.500)	(0.132)	(0.359)	(0.411)	(0.281)	(0.112)	(0.266)
				[-0.141]	[-0.406]	[-0.533]	[0.118]	[0.097]	[0.426]
Post-policy (P16–30)	12.149 ***	13.120 ***	17.213 ***	-0.709 ***	-1.721 ***	-1.591 ***	1.906 ***	-0.025	1.125 ***
	(2.574)	(3.641)	(4.223)	(0.266)	(0.535)	(0.416)	(0.400)	(0.143)	(0.166)
				[-0.238]	[-0.467]	[-0.427]	[0.154]	[-0.004]	[0.206]
Period	-0.691 ***	-0.604 ***	-0.722 ***	0.063 ***	0.097 ***	0.101 ***	-0.085 ***	0.011	0.005
	(0.176)	(0.171)	(0.146)	(0.010)	(0.023)	(0.016)	(0.018)	(0.014)	(0.007)
				[0.022]	[0.028]	[0.026]	[-0.011]	[0.002]	[0.001]
Constant	3.886	11.129 *	9.279	-0.577	-1.968 ***	-0.751	-1.574 ***	-0.429	-0.685
	(5.492)	(6.369)	(21.171)	(0.548)	(0.587)	(1.610)	(0.382)	(1.218)	(1.542)
Observations	1200	1200	1200	1200	1200	1200	1200	1200	1200
Wald test: Policy = Post-	*p*<0.01	*p*<0.01	*p*<0.01	*p*<0.01	*p*<0.01	*p*<0.01	*p*<0.01	*p*<0.01	*p*<0.01
F test: 3 policies	(*χ*^2^(2)=9.76 *p*=0.01)			(*χ*^2^(2)=21.96 *p*<0.01)			(*χ*^2^(2)=33.58 *p*<0.01)		
F test: 3 post-policies	(*χ*^2^(2)=1.14 *p*=0.57)			(*χ*^2^(2)=5.24 *p*=0.07)			(*χ*^2^(2)=42.69 *p*<0.01)		
F test: 3 constants	(*χ*^2^(2)=0.81 *p*=0.67)			(*χ*^2^(2)=3.31 *p*=0.19)			(*χ*^2^(2)=1.13 *p*=0.57)		

Notes: Standard errors are in parentheses and clustered by group. Marginal effects of Probit regressions are in brackets. Regressions include controls for gender, age and if the participant studies economics.

* *p* < 0.1;

** *p* < 0.05;

*** *p* < 0.01

Fig 2 also shows that there are many zero and full contributions in the *Post-policy* phase in the *Monetary Punishment* treatment. We further examine these contributions with the help of Probit regressions, taking zero or full contributions as the dependent variable in each treatment condition. Results in Table 3 reveal that all three incentives reduce the level of zero contributions and increase the number of full contributors. However, although *Non-monetary Reward* appears to be efficient in reducing the number of free-riders, it is less efficient in increasing the proportion of full-contributors. Also, during the *Post-policy* phase, *Non-monetary Reward* does not increase the proportion of full contributors compared to the *Pre-policy* phase. This probably explains why we observe lower average contributions in this treatment during the policy phase.

**Result 1**. *All incentive mechanisms significantly increase contributions during the Policy phase compared to the Pre-policy phase. The highest level is observed in Monetary Punishment followed by Non-monetary Reward and Recommendation*.

### Individual preferences

Finally, we turn to individual preferences for contributions. As explained in the design section, we elicited the individual preferences using a public goods game played in the Strategy Method, as proposed by [22]. We are interested in seeing how the subjects’ preferences explain the contribution behavior and also how they are affected by the incentives over time.

We rely on the first Strategy Method, and we classify subjects according to their decisions, as explained in Section. Table 4 presents the distribution of types in each treatment condition. Overall, we have 45 strong CCs (37.5%), 36 weak CCs (30%), 26 free-riders (21.7%), and 13 subjects that we classify as “other” types (10.8%). These percentages are in line with previous studies [22, 47]. The proportion of each type does not significantly differ in the three treatment conditions (*χ*^2^(6, 120)=2.45 *p*=0.87). The S2 Appendix contains supplementary results on the validity of the criteria for our classification.

**Table 4:** Type classification.

	All	Reco	Reward	Punish
Strong CC	45 (37%)	15 (38%)	14 (35%)	16 (40%)
Free-rider	26 (22%)	7 (18%)	9 (23%)	10 (25%)
Weak CC	36 (30%)	13 (33%)	14 (35%)	9 (23%)
Other types	13 (11%)	5 (13%)	3 (8%)	5 (13%)

**Fig 3:**
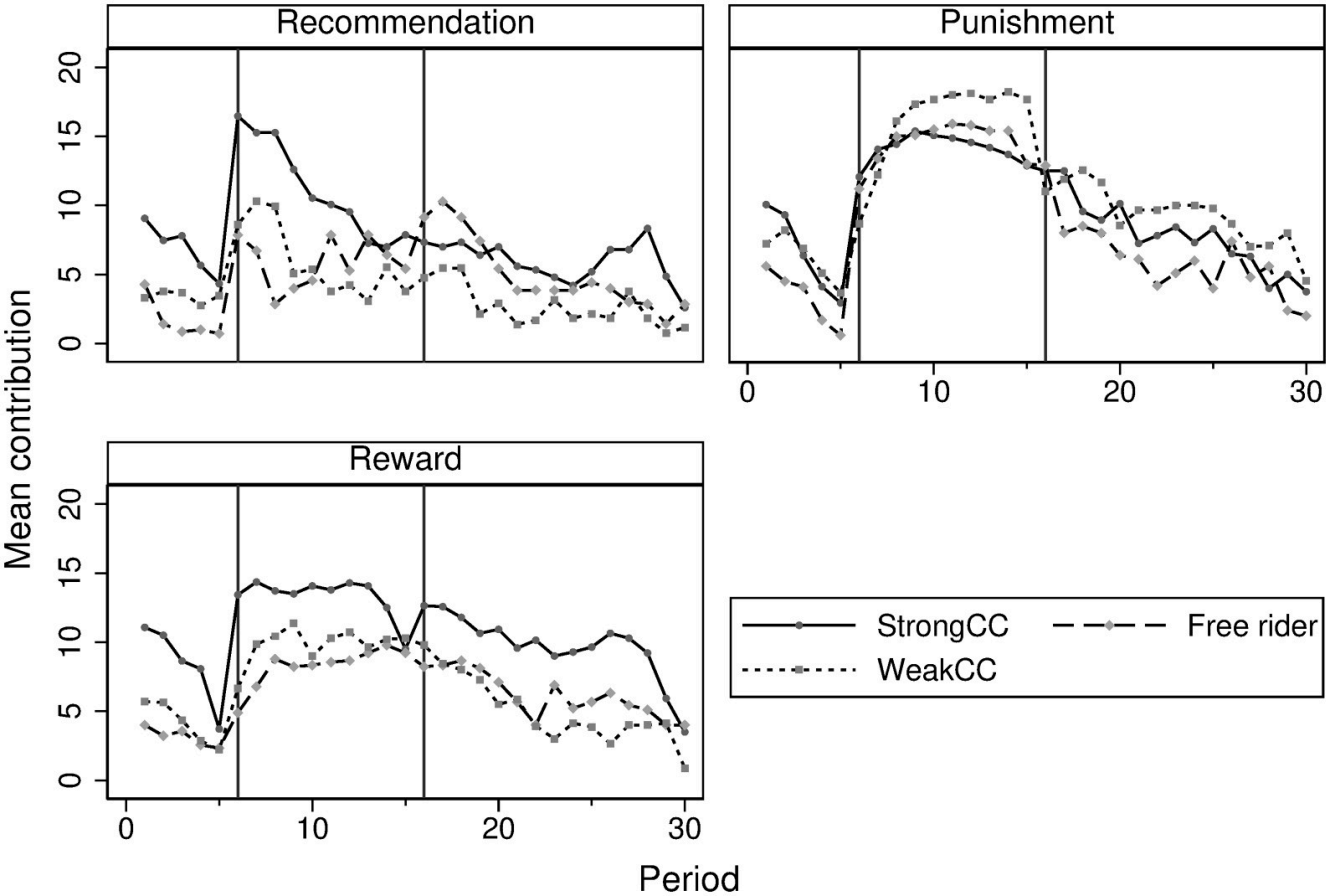
Average contributions in the PGG.

Table 5 shows the average contributions for each type of subject and during each phase in the three treatments. Strong CCs generally contribute more than the weak CCs and free-riders, except in the *Policy* phase in *Monetary Punishment*. In the *Policy* phase, as expected from Prediction 2a, participants’ preferences do not matter for *Monetary Punishment*. The fact that all types react in a similar way in relation to *Monetary Punishment* is shown by a non-parametric test that takes individual averages as the unit of observation. The Kruskal-Wallis test is used (*Punishment* in the *Policy* phase, including all four types): *χ*^2^(3, 400)=3.70 *p*=0.30). This is not the case for *Recommendation* and *Non-monetary Reward* where we observe differences between types of participants. Results from post-hoc comparison tests suggest that in both *Recommendation* and *Non-monetary Reward*, strong CCs contribute significantly more than the other two types (Dunn’s z-test statistics with Bonferroni adjusted p-values for multiple comparisons). Strong CC vs. Free-rider: (*Recommendation*) z = 4.93 *p*<0.01, (*Reward*) z = 5.60 *p*<0.01. Strong CC vs. Weak CC: (*Recommendation*) z = 5.41 *p*<0.01, (*Reward*) z = 4.55 *p*<0.01. Free-rider vs. Weak CC: (*Recommendation*) z=-0.44 p = 1.00, (*Reward*) z=-1.57 *p*=0.35). Therefore, our Prediction 2b that strong conditional cooperators contribute the most is confirmed. We do not find the evidence about differences between free-riders and weak CCs in *Recommendation* and in *Non-monetary Reward*. This implies that free-riders contribute more than what their type would initially predict. Weak CCs decrease the contributions due to the introduction of these two treatments. The results can also be graphically confirmed in Fig 3, which displays the average contributions for each type in each treatment condition.

**Table 5:** Individual average contribution for each classification.

	Pre-policy (Periods 1–5)		Policy (Periods 6–15)		Post-policy (Periods 16–30)	
	Mean	Std.Dev.	Mean	Std.Dev.	Mean	Std.Dev.
Recommendation						
Strong CC	6.87	4.60	11.19	7.72	5.97	6.97
Free-rider	1.66	3.77	5.89	7.49	5.03	7.07
Weak CC	3.40	3.63	5.97	6.59	2.69	4.43
Other types	7.96	4.74	12.82	5.71	8.99	6.37
Non-monetary Reward						
Strong CC	8.40	7.78	13.32	6.73	9.72	7.44
Free-rider	3.13	4.99	8.24	7.05	6.19	7.84
Weak CC	4.16	3.48	9.84	6.02	5.03	6.43
Other types	3.73	3.86	10.70	3.04	3.11	3.94
Monetary Punishment						
Strong CC	6.56	5.58	14.12	6.86	7.89	9.02
Free-rider	3.30	5.75	14.57	6.32	6.09	8.48
Weak CC	6.22	4.73	16.17	5.75	9.34	8.97
Other types	4.16	4.62	14.62	5.21	6.81	8.55

Unit(token)

**Result 2**. *During the Policy phase, Strong CCs sustain the highest contribution in Recommendation, as predicted, but the level is lower than for the other two treatments. In Non-monetary Reward, the highest contribution is observed for Strong CCs and the lowest one for free-riders. Participants’ preferences matter less for Monetary Punishment since all types react to the incentive in a similar way*.

As opposed to Periods 6–15, test results show a difference between the types of subjects for all treatment conditions in Periods 16–30 (Kruskal-Wallis test, including all four types: (*Recommendation*) *χ*^2^=57.15 *p*<0.01, (*Reward*) *χ*^2^=58.04 *p*<0.01, (*Punishment*) *χ*^2^=13.35 *p*<0.01). A post-hoc comparison in each treatment condition then suggests a similar pattern in *Recommendation* and *Non-monetary Reward* that Strong CCs sustain the highest contribution (Dunn’s test z-test statistics with Bonferroni adjusted p-values for multiple comparisons. Strong CC vs. Free-rider: (*Recommendation*) z = 2.40 *p*=0.05, (*Reward*) z = 5.20 *p*<0.01. Strong CC vs. Weak CC: (*Recommendation*) z = 4.71 *p*<0.01, (*Reward*) z = 6.84 *p*<0.01. Free-rider vs. Weak CC: (*Recommendation*) z = 1.47 *p*=0.42, (*Reward*) z = 0.85 p = 1.00. Although the high mean contribution between free-riders and weak CCs is reversed this time compared to that of the *Policy* phase, we find no statistical difference between these two types of participants. In *Monetary Punishment*, all types react similarly during the *Policy* phase, whereas in the *Post-policy* phase, weak CCs show the highest contribution (Dunn’s z -test statistics with Bonferroni adjusted p-values for multiple comparisons. Strong CC vs. Free-rider: (*Punishment*) z = 1.81 *p*=0.21. Strong CC vs. Weak CC: (*Punishment*) z=-2.45 *p*=0.04. Free-rider vs. Weak CC: (*Punishment*) z=-3.81 *p*<0.01). Tests reveal no evidence of differences between free-riders and strong CCs.

Finally, we investigate how the effect of turning incentives on and off over time differs across agent types through regression analysis. Results are shown in Table 6. The reference categories of type classification and sequence dummies are strong CCs and *Pre-policy* phase, respectively. Since our focus is not on other types, we exclude the results for this type from the table, even though they are included in the regression analysis.

**Table 6:** Determinants of individual contributions.

Dependent variable: Individual contribution			
	Tobit estimation		
	Recommendation	Reward	Punish
Free-rider	-12.178 **	-7.399 **	-12.495 ***
	(5.184)	(3.337)	(2.809)
Weak CC	-5.506 ***	-3.840	-4.172
	(2.070)	(2.471)	(2.683)
Policy (P6–15)	10.618 ***	12.325 ***	19.276 ***
	(3.095)	(2.475)	(6.198)
Post-policy (P16–30)	10.771 ***	14.130 ***	13.812 **
	(3.392)	(3.822)	(6.636)
Free-rider * Policy	2.773	0.180	13.466 ***
	(3.553)	(2.563)	(4.542)
Free-rider * Post-	8.670 **	1.713	7.934
	(4.276)	(3.112)	(6.686)
Weak CC * Policy	-2.736	-0.499	4.919
	(3.344)	(1.514)	(4.120)
Weak CC * Post-	-0.748	-3.088 **	6.004
	(3.893)	(1.482)	(6.385)
Period	-0.688 ***	-0.602 ***	-0.720 ***
	(0.176)	(0.169)	(0.142)
Constant	5.851	11.961	8.650
	(4.200)	(8.482)	(19.793)
Observations	1200	1200	1200
Wald test			
FR = Weak CC	*p*=0.02	*p*=0.09	*p*<0.01
Policy = Post-	*p*<0.01	*p*<0.01	*p*=0.01
FR*Policy = FR*Post-	*p*=0.01	*p*=0.76	*p*<0.01
F test: 3 FR*Policy	(*χ*^2^(2)=6.99 *p*=0.03)		
F test: 3 FR*Post-	(*χ*^2^(2)=2.16 *p*=0.34)		

Standard errors are in parentheses and clustered by group. Regressions include controls for gender, age, if the participant studies economics and “Other” type as one of the classification.

* *p* < 0.1;

** *p* < 0.05;

*** *p* < 0.01

In order to see how each type is affected by the presence or absence of incentives over time, we need to look at type classification dummies and interaction terms with sequence dummies in Table 6. In *Recommendation*, the free-rider coefficient shows that the contribution of free-riders is significantly lower than that of strong CCs in the *Pre-policy* phase. Also, looking at the interaction terms and isolating the effects of being in the *Post-policy* phase for each type of subject, we observe that the average contribution of free-riders in the *Post-policy* phase is higher than that of weak CCs and a little lower than strong CCs. Comparing with the *Pre-policy* phase and using the strong CC’s contribution in the *Pre-policy* phase as a reference point, the difference of contributions for free-riders turns from negative to positive between the *Pre-policy* and *Post-policy* phases. The difference between the two phases for the free-riders may also be explained by the spike we observe in the first periods of the *Post-policy* phase. This high effect on the free-riders can be related to two elements. First as shown in Chaudhuri and Paichayontvijit (2017), *Recommendation* helps to sustain cooperation at high level. Second it appears that precisely saying that contributing is for the sake of the subjects helps to fix cooperation. This because the group members realize that it is mutually beneficial to contribute. As for *Non-monetary Reward*, weak CCs reduce their contribution between the *Pre-policy* and *Post-policy* phases, which is shown by the insignificance of weak CC coefficient for the difference between weak CCs and strong CCs in the *pre-policy* phase and the negative sign condition of the weak CCs’ interaction term with *Post-policy*. Finally, in *Monetary Punishment*, free-riders contribute less than strong CCs in the *Pre-policy* phase, but considerably increase their contribution in the *Policy* phase.

**Result 3**. *A closer look at individual preferences reveals that contribution patterns vary from treatment to treatment in the Policy phase. Strong CCs continue to make the highest contribution in Recommendation, but it is free-riders that increase the contribution compared to the Pre-policy phase. Recommendation appears to have stronger persistent effects than the other two treatments*.

## Discussion and conclusion

In this study, our aim is to compare the effects of three different incentives to contribute in a repeated public goods game with a fixed partner design. We are particularly interested in explaining the drivers of the differences we observe in the short and long terms. Our findings shed light on the importance of heterogeneous preferences, not only among agents but also in each individual over time, and beliefs about others’ decisions to understand the contribution behaviors.

First, the average contributions are high in response to the beginning of all treatment conditions, but the magnitude is different in each incentive. Contrary to our expectations, the *Monetary Punishment* treatment works most effectively during periods in which subjects can punish other group members. In general, the *Recommendation* treatment has the lowest contribution. Once the incentives are removed, all the treatments have the long-lasting effects, although their impacts appears to be similar when looking at group averages. This implies that the persistence of the effect in the post-intervention phase is weaker in the *Monetary Punishment* treatment than those of *Non-monetary Reward* and *Recommendation*.

Second, the classification of individuals according to their initial preference allows us to delve deeper into the determinants of the voluntary contribution. The *Monetary Punishment* treatment appears to successfully enhance the contribution of all types of subjects in the *Policy* phase, and its effect on free-riders is greater than in the other two treatments. The *Non-monetary Reward* treatment turns out to backfire against weak CCs between the *Pre-policy* and *Post-policy* phases. It is surprising that *Monetary Punishment* does not have adverse effects; only *Non-monetary Reward* does. Hence, our experiment suggests that treatments may have a negative impact when efforts to reward others do not pay off, rather than when costly actions to punish others do not pay off. Despite the fact that the *Recommendation* treatment makes free-riders increase their contribution over time even after the withdrawal of incentive, it ends up with low contribution levels across treatments. One of the reasons for low impact of *Recommendation* may come from the repetition of the message presented in each period. Subjects, except for free-riders, could be exhausted, although punishing or rewarding others is more burdensome than receiving the informational nudge. [33] repeat the recommendation message every four rounds and it has a higher efficacy than the punishment. Taken together with the results of our experiments, this suggests that, depending on the intervention and its frequency, *Recommendation* may be a mechanism that encourages the emergence of cooperative behavior between free-riders; by actually changing their behavior.

Finally, our experiment has limitations in its design due to the situation around the practical implementation of agricultural environmental policies. One is related to that the social interactions within the group. It is given by information about others’ contributions. The way of interacting with others in the real world is probably more descriptive, including discussions about information they receive. Interpersonal communication may boost the incentive effects, as mentioned in [73] concerning their experiment on PES. Furthermore, we do not reflect the possibilities of long-term investments for subjects, such as the carry-over of their tokens to the following periods or the investment irreversibility, which may increase the contribution [74, 75]. In practice, PES or AES may provide opportunities for participants to invest in long-term capital such as machinery equipment or to acquiring further expertise [19]. Moreover, synergistic effects that a combination of incentives has a greater impact than the sum of the individual effects can be explored further [5, 51, 76]. Hence, these can be a possible extension of our experiment.

The analysis of heterogeneous preferences and beliefs of agents is of value for a public policy with long-lasting effects. As previously mentioned, real farmers participating in the French AES scheme are mostly conditional cooperators [19], and for the conservation program to work, the proportion of different types needs to be taken into account [42]. By performing the Strategy Method twice, we confirm that individuals’ stated preferences between ex-ante and ex-post PGG do not change much. However, no study on the temporal change in individual preferences has yet to be conducted in the field. In addition, we have not fully explained the long-lasting effects such as why free-riders in *Recommendation* increase their contribution in the post-intervention periods. It is always challenging to show the exact magnitude of each determinant for longevity, especially behavioral factors, since it may be difficult to detect but necessary as a component of interactions with others. In our experiment, we distinguish between *Non-monetary Reward* and *Recommendation*, but both are non-monetary incentive mechanisms in the broad sense. We expect that the comprehensive *Recommendation* treatments would lead to a visible impact and serve as a cost-effective policy instrument. Thus, it would be interesting to look further into the interrelation and dynamics of the incentives and various agents to boost collective decision-making. Concerning the careful policy design, our findings stress the need for maintaining the contribution level of conditional cooperators with a more comprehensive informational nudge at the same time, while waiting for an increase in free-riders’ contributions at a later stage.

Among the recommendations that can be drawn from these results, the first is that not all individuals in a society can be involved and solicited in the same way: both their preferences and their interactions with others must be taken into account. Moreover, on a societal problem that involves a long period of time, there are solutions that are less costly than others (Recommendation here) and that allow the involvement of even those who were not initially involved.

At present, we situate ourselves at the standpoint of experimental economics. However, the long-lasting effects in environmental policies involve numerous components, e.g., the external effect of climate change or natural recovery and the involvement of agents over time. It might be possible that one-shot intervention, e.g., an afforestation project, does not require a long-term commitment on the grounds of spontaneous vegetation growth, yet is still linked to biodiversity conservation in the long run. Interdisciplinary research that takes both economics and ecological points of view into account is therefore necessary to unravel the long-lasting effects of incentives.

## Supporting information

S1 Appendix Instruction (Monetary punishment). (PDF)

S2 Appendix Validity of classification criteria [21, 77]. (PDF)

S3 Appendix Additional figures. (PDF)
