# Supplementary material for: Long-lasting effects of incentives and social preference: A public goods experiment
Source: PLoS One. 2022 Aug 25;17(8):e0273014. doi: 10.1371/journal.pone.0273014 (PMC9409558; doi:10.1371/journal.pone.0273014)
Supplement: S1 Appendix — (PDF) [file pone.0273014.s001.pdf]

Appendix A Instruction (Monetary Punishment)

Thank you for participating in this experiment on decision-making. In this experiment, your payoff depends on your decisions and those of other participants. We therefore ask you to read these instructions carefully. They should help you to understand the experiment. All your decisions are anonymous. You will never enter your name on the computer. You will indicate your choices on the computer in front of you.

From now on we ask you not to speak any more. If you have a question raise your hand and an experimenter will come and answer you in private. It is strictly forbidden to communicate with another participant during the experiment. If you do not respect this rule you will be excluded from the experience and from any payment.

You will take part in four independent tasks. Here are the instructions for the first task; the next ones will be given to you later.

TASK 1

During this task, you will be able to accumulate payoffs expressed in tokens. At the end of the experience, your total accumulated token winnings will be converted into euros at the following rate; 10 tokens = € 1.

Participants in this experiment are randomly divided into groups of 4, so you are in a group with three other participants and none of you can know the identity of the other members of your group or the composition of the other groups.

At the beginning of this task you are given 20 tokens and you have to decide how to use them. Specifically, you have to decide how many tokens you want to contribute to the common project of the group that you are a member of and how many tokens you want to keep for yourself. The choice of your contribution to the project automatically determines how many tokens you keep for yourself (20 tokens minus your contribution).

Each member of the group makes the same decision and the contributions to the common project give the right to an earning. Each token paid into the project brings in 0.4 tokens to each member of the group.

- For example, if the total contributions of the four group members is 60 tokens, each group member receives 0.4*60=24 tokens. If the total contribution is 15 tokens, each group member receives an income of 0.4*15=6 tokens from the project.

Your earnings are therefore the sum of two amounts:

1. The number of tokens you have kept for yourself.

2. The income obtained through contributions to the common project

Your earnings = (20 - your contribution to the project)
+ 0.4 * (the total of the group members' contributions)

In the course of this task, you will have to make two types of decisions:

A) You must choose your contribution, between 0 and 20, to the joint project.

B) You then fill in a contribution table. In this table, you have to give the number of tokens you want to contribute to the joint project for each average contribution made by the three other group members (rounded up to the nearest unit). You must enter an amount between 0 and 20; that is your contribution to the project if the other three contributed an average of 0 token, if they contributed 1 token, if they contributed 2 tokens, etc.

Average contribution of the three other members	0	1	2	3	4	5	6	7	8	9	10
Your contribution											
Average contribution of the three other members	11	12	13	14	15	16	17	18	19	20	
Your contribution											

Once all of the participants have made their two decisions, A and B, one member from each group will be chosen at random. For this member, the earnings will be determined according to the contribution table completed in B. For the other members, the winnings will be determined by decision A. Since you do not know who will be selected in advance, be careful to make your decisions in both cases: A and B. These two decisions can be decisive for your earnings. The following example should clarify the calculation of earnings:

- Example: If you are chosen by the computer, your earnings will be determined by the contribution table. For the other three members of your group, their earnings will be calculated according to their decision in A. Suppose they have contributed 6, 3 and 9 tokens, respectively. The average contribution of these three participants is therefore 6 tokens = (6+3+9)/3. If you have indicated in the contribution table that for an average contribution of 6 tokens from the other members of your group, you wish to contribute 7 tokens, then the total contribution to the joint project is 6 + 3 + 9 + 7 = 25 tokens. All the members of the group to which you belong then receive an income of 0.4*25=10 tokens in addition to the tokens (out of the 20 initial endowment) that they have not contributed to the project and kept for themselves. The total earnings for the first member are 20-6+10=24 tokens. The second member receives 20-3+10=27 tokens. The third member receives 20-9+10=21 tokens and you receive

20-7+10=23 tokens. If instead of 7 tokens you have indicated in the contribution table that you want to contribute 2 tokens when the other three contribute an average of 6 tokens, the total contribution is 6 + 3 + 9 + 2= 20 tokens. All members of the group receive an income of 0.4*20=8 tokens from the joint project. Your total earnings are then 20-2+8=26 tokens.

The decision as to whether it is A or B and the associated gain will be communicated to you at the very end of the experiment.

TASK 2

This task consists of an experiment that comprises five periods. You are again divided into a group of four players chosen at random. The composition of the group will remain the same over the five periods.

This game works similarly to Task 1. You receive 20 tokens at the beginning of each period, and you decide how many tokens you will contribute to a common project of the group of which you are a member and how many tokens you will keep for yourself.

Each member of the group makes the same decision, and contributions to the common project give the right to an earning. Each token paid into the project brings in 0.4 tokens for each member of the group. Your earnings in each period are therefore calculated as follows:

Your earnings = (20 - your contribution to the project)<br>+ 0.4 * (the total of the group members' contributions)

For this task, 50 tokens are worth € 1. In each of the five periods of this task, you will have to make two decisions:

1. First, you will have to decide on your contribution to the project. This decision is similar to decision (A) made in Task 1. In this task, you do not have to fill in a contribution table; you simply have to indicate how much you want to contribute to the common project.

2. You must then estimate the average contribution to the project (in whole numbers) of the other three members of your group for the period. The closer your estimate is to the actual average contribution, the higher your earnings will be:

   - If your estimate is correct (and corresponds exactly to the actual average contribution) 3 tokens will be added to your final payoff.

   - If your estimate deviates by 1 token from the actual estimate, 2 tokens will be added to your final payoff.

- If your estimate deviates by 2 tokens from the actual estimate, 1 token will be added to your final payoff.
- If your estimate deviates by 3 or more tokens, no token will be added.

In each period, your payoffs are therefore the sum of two amounts: the earning obtained by using your initial allocation of 20 tokens and the earning obtained if you have more or less correctly assessed the average contribution of the other three members of the group. At the very end of the experiment, the sum of your earnings obtained during the five periods will be paid to you and will be added to the earnings of Task 1.

TASK 3

This task consists of 25 successive periods divided into two distinct parts: a first part of 10 periods followed by a second part of 15 periods. You are part of the same group of four players, like in Task 2. The group remains the same throughout the task. The game works in the same way as in Task 2. For this task, 50 tokens are always worth € 1.

Periods 1-10

Each of the 10 periods consists of two stages.

Stage 1

You have to decide how many tokens you want to contribute to the common project. You also need to estimate the average contribution of the three other members of the group.

Stage 2

At the beginning of the second stage, you are informed of the level of individual contributions that each member of your group has made to the project. You then have the opportunity to reduce the income of each group member by distributing points. You can distribute a large number of points to a member of your group if you disapprove of their investment decision in the first step. You can give between 0 and 10 points maximum: 10 points if you strongly disapprove of their decision and 0 points if you do not disapprove of their decision. Each disapproval point reduces the income obtained in the first step by 1 token.

Other members of your group can also reduce your Stage 1 earnings if they wish. You must therefore decide for each member of your group, after learning about their investment in the common project, how

many disapproval points you want to give them. If you do not want to change a member's earnings, you can assign 0 disapproval points.

Remember that you and the same three participants will form the group until the end of this task. However, you will not be able to identify each member individually. In each period, the investments made in the joint project will be presented to you in ascending order without any indication of the link between an investment and the group member who made it. Each item you give to another member of your group has a cost to you. Each point you give reduces your Step 1 earnings by 0.25 tokens.

- If you distribute 2 points to a member of your group. his earnings are reduced by 2 tokens and yours are reduced by 0.25 * 2 = 0.5 tokens. If you give 8 extra tokens to another member of your group, their earnings are reduced by 8 tokens but your earnings are reduced by a total of (2+8) * 0.25 = 2.5 tokens.

- If you give 0 points to a member of your group, neither their earnings nor yours are affected.

The total amount of your winnings at the end of the period is calculated as follows:

Payoff for the period = gains from Step 1<br>- sum of points received from other members<br>- 0.25 * points distributed to other members

Your earnings for the period may therefore be negative if your Step 1 earnings are not sufficient to offset the points received and the cost of points distributed to other members. Once all participants have made their choice, your earnings for the period will be communicated to you and another period will begin.

Periods 11-25

In periods 11 to 25, you will have to make the same decisions as in Step 1 of periods 1 to 10. However, you will no longer have the opportunity to distribute disapproval points to your group members. The other members of your group will not be able to distribute points either. Therefore, you will no longer be able to reduce the earnings of your group members, just as no member of your group will be able to reduce your earnings.

Your only decisions, in each period, will be how much of your 20 initial endowment tokens you want to contribute to the common project and how much you think the three other group members will contribute on average. The return on the common project is the same as in the first 10 periods.

TASK 4

This last task is identical to Task 1. You are divided into new randomly selected groups of four. You have to decide how much to contribute in decisions (A) and (B). The value of the tokens earned is the same as Task 1: 10 tokens are worth € 1.
