# Supplementary material for: Long-lasting effects of incentives and social preference: A public goods experiment
Source: PLoS One. 2022 Aug 25;17(8):e0273014. doi: 10.1371/journal.pone.0273014 (PMC9409558; doi:10.1371/journal.pone.0273014)
Supplement: S2 Appendix — (PDF) [file pone.0273014.s002.pdf]

Appendix B Validity of Classification Criteria

Fig 4 illustrates the average conditional contributions of each type of strong conditional cooperators (Strong), free-riders (FR), weak conditional cooperators (Weak), and others (OT) from the 1st SM.

Fig 4. Average Conditional Contributions in the 1st SM

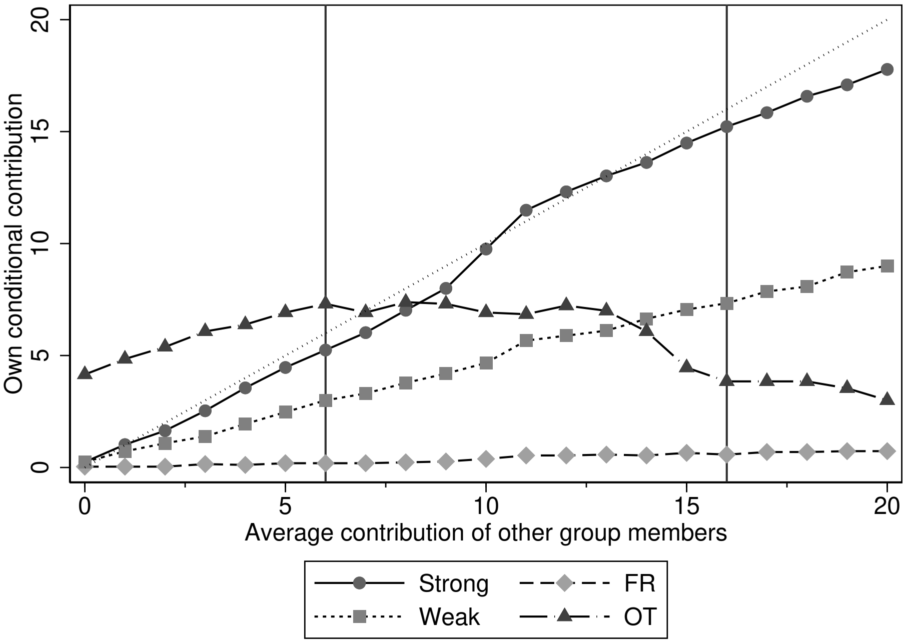

Our classification criteria are largely consistent with the level of contribution at the first period for each type. Provided that the contribution behavior of other types is hard to anticipate, strong CCs should be, in principle, the highest contributors, followed by weak CCs. In our results shown in Table 7, this is true except for *Recommendation*. The average contribution of weak CCs in *Recommendation* is lower than that of free-riders. As Fig 5 shows, the average in the 1st SM for *Recommendation*'s weak CCs is not substantially different from those who in the other two treatments, so it is less likely that the low contribution at the initial period comes from the inappropriate classification criteria. Moreover, when we look at their average for the *Pre-policy* phase in Table 5, it is higher than that of free-riders. Therefore, the weak CCs in *Recommendation* may be confused at the first period when the interaction with others begins, although they are immediately returns to the behavior that corresponds to their initial preferences. In relation to free-riders in all of the treatment conditions, they contribute more than 10% of their endowment, which is a deviation from the initial preferences. However, this tendency is reported in the literature [21, 77].

Fig 5. Average Conditional Contribution in the 1st SM

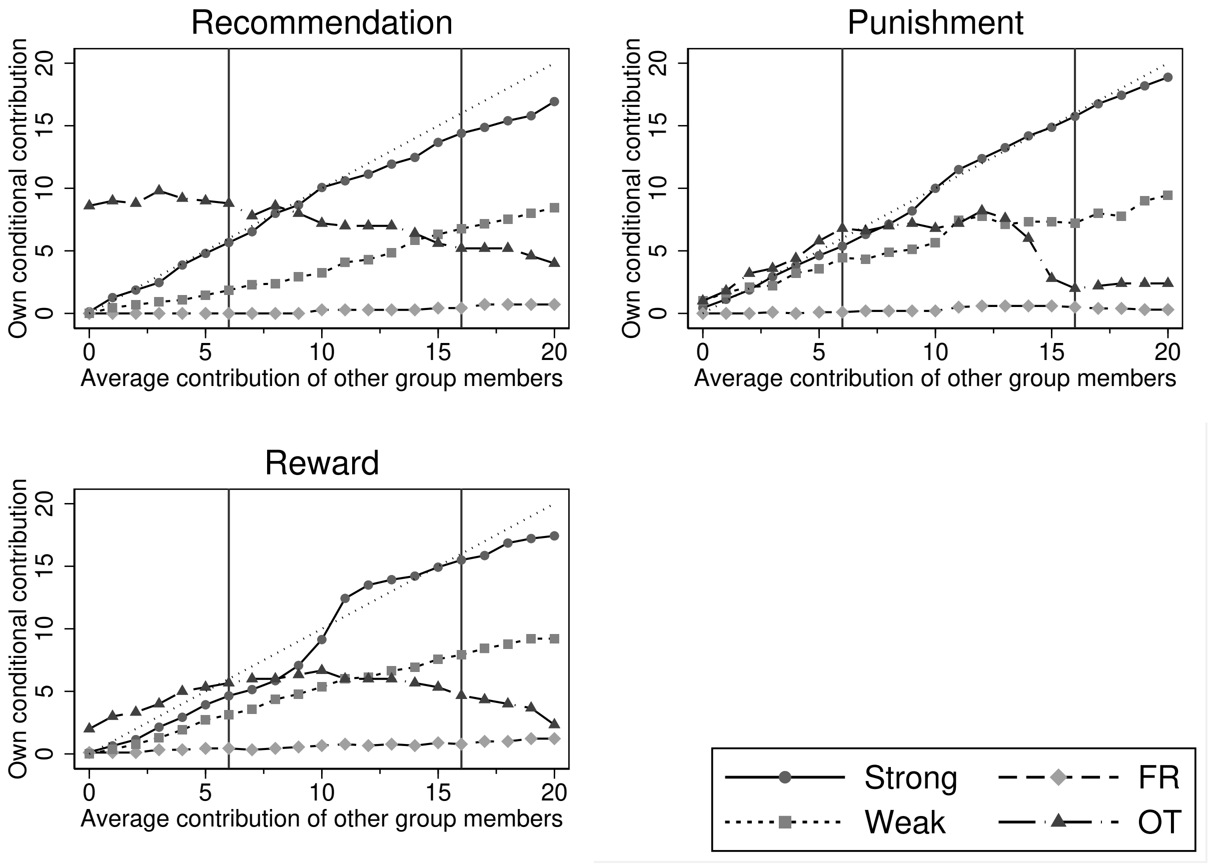

Table 7. Average Contributions at the 1st period

	All			Recommendation			Non-monetary Reward			Monetary Punishment		
	Mean	Std.Dev.	Obs	Mean	Std.Dev.	Obs	Mean	Std.Dev.	Obs	Mean	Std.Dev.	Obs
Strong CC	10.04	5.92	45	9.07	4.04	15	11.07	8.30	14	10.06	5.08	16
Free-rider	4.69	6.72	26	4.29	7.32	7	4.00	6.26	9	5.60	7.31	10
Weak CC	5.22	3.85	36	3.31	3.79	13	5.71	3.12	14	7.22	4.06	9
Other types	7.77	4.48	13	9.20	4.66	5	7.00	4.36	3	6.80	4.97	5

Unit(token)
