# Supplementary figures and images for: Long-lasting effects of incentives and social preference: A public goods experiment
Source: PLoS One. 2022 Aug 25;17(8):e0273014. doi: 10.1371/journal.pone.0273014 (PMC9409558; doi:10.1371/journal.pone.0273014)

## Appendix C Additional Figures

**Fig 6.** Contribution and Belief

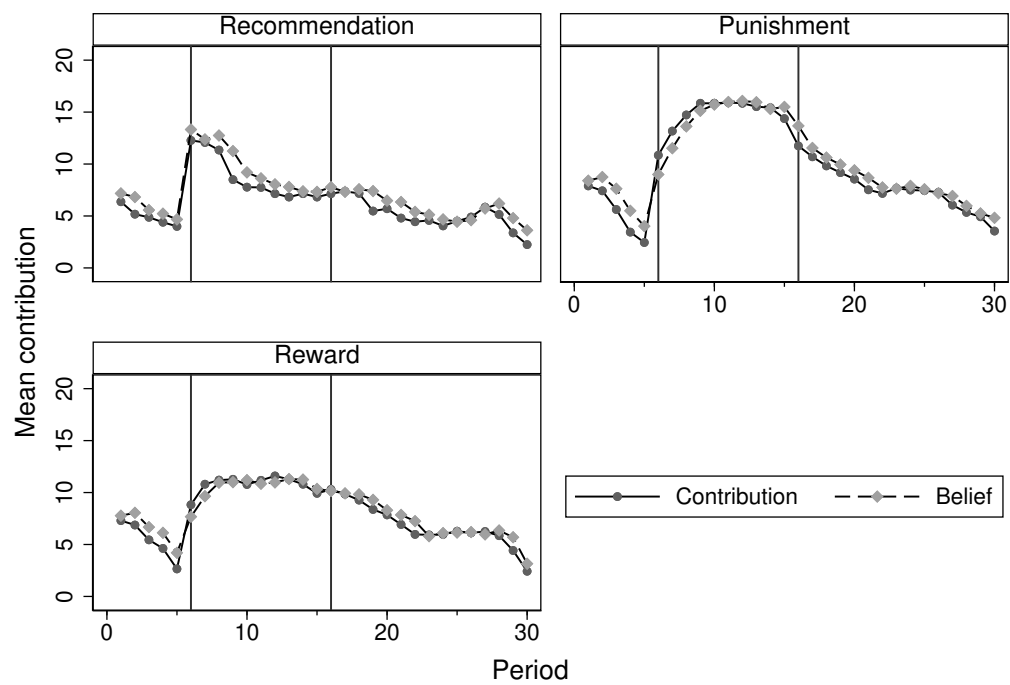

Supplement: S3 Appendix — (PDF) [file pone.0273014.s003.pdf]
